# Inclusion of sex and gender in biomedical research: survey of clinical research proposed at the University of Pennsylvania

**DOI:** 10.1186/s13293-017-0139-5

**Published:** 2017-06-21

**Authors:** Anne Freeman, Patrick Stanko, Lily N. Berkowitz, Neanta Parnell, Anastasia Zuppe, Tracy L. Bale, Tracy Ziolek, C. Neill Epperson

**Affiliations:** 1 Department of Psychiatry, Perelman School of Medicine at the University of Pennsylvania, Philadelphia, PA USA; 2 Department of Obstetrics and Gynecology, Perelman School of Medicine at the University of Pennsylvania, Philadelphia, PA USA; 3 Penn PROMOTES Research on Sex and Gender in Health, University of Pennsylvania, 3535 Market Street, Rm 3001, Philadelphia, PA 19104 USA; 4 Institutional Review Board, University of Pennsylvania, Philadelphia, PA USA; 5 Neuroscience Center, School of Veterinary Medicine, University of Pennsylvania, Philadelphia, PA USA

**Keywords:** Sex, Gender, Research, Informed consent, Ethics, Research, Institutional review board

## Abstract

**Background:**

The 2015 National Institutes of Health (NIH) policy that sex be considered as a biological variable (SABV) is now a critical part of the peer-review process for NIH funding as well as publication in several high-impact scientific journals. We sought to determine the degree to which biomedical researchers at the University of Pennsylvania already consider SABV or gender in their research.

**Methods:**

We reviewed 240 research protocols approved by the University of Pennsylvania Investigational Review Board (IRB) consecutively submitted between January and July 2016. Each protocol was searched for the terms sex, gender, male, female, man, and woman and justifications related to the population under study. A PubMed search was conducted to determine the current state of knowledge regarding potential sex and/or gender differences with respect to protocol topic. Data were summarized using descriptive statistics.

**Results:**

Of the 165 (68.8%) protocols that included one of the search terms, only 24 (14.5%) provided justification for the choice of the sex/gender of the population studied. Sixty-three percent (*n* = 151) of the protocols focused on topics for which the extant literature supports at least a moderate degree of sex/gender differences in some aspect of the disorder/condition being studied. Of these, only three (2.0%) indicated that the investigator would consider sex or gender impact on their primary outcomes.

**Conclusions:**

Review of a subset of IRB protocols submitted at a major research institution suggests that very few investigators are considering sex or gender as important variables in their clinical research at the stage of protocol development. IRBs are in an excellent position to encourage investigators to consider SABV and gender in order to enhance the rigor of research design, maximize the importance of the resulting knowledge, and ensure that subject selection is equitable. These findings serve as the basis for developing an intervention at the level of IRB protocol development and submission that will promote consideration of SABV and/or gender, factors with critical import to patient safety and efficacy of interventions.

## Background

The vast majority of medical conditions impacting human health are characterized, in part, by pronounced sex differences in age of onset, phenomenology, identification, treatment uptake, and adherence, as well as treatment efficacy and adverse effects of interventions. Until the later decades of the twentieth century, women of reproductive capacity were considered a vulnerable population and systematically excluded from clinical research. This policy upheld the male norm or “the tendency to use males as the standard and to see females as deviant or problematic, even in studying diseases that affect both sexes” and was justified as a protectionist measure [1].

Without inclusion of sufficient numbers of both males and females, consideration of potential sex differences in efficacy and safety of medical interventions was impossible and has levied a disproportionate risk upon women. For example, in 1989, the Physicians’ Health Study observed the effects of aspirin in over 20,000 male physicians. In the male study, aspirin was found to lower the risk of myocardial infarction with a slightly increased risk of stroke [2]. In 2005, a study on the effects of aspirin was conducted in a sample of 39,000 female participants. Findings were considerably different for females, with aspirin reducing the risk of stroke and but having no significant reduction in risk for myocardial infarction or death from cardiovascular disease [3].

When sex is accounted for as a variable, females tend to have a more adverse effect to prescription medications. Out of the ten prescription drugs removed from the US market between 1997 and 2001, eight were found to have had greater adverse effects in women [4]. Still, as recently as 2013, the FDA approved labeling changes for dosing of zolpidem, a drug approved for the short-term treatment of insomnia decades earlier. The recommended starting dose was reduced in women, as metabolism of the drug is slower in females, leading to higher drug concentrations and morning drowsiness in women compared to men [5].

Since the early 1990s, there have been numerous efforts on the part of governmental research and funding agencies to promote greater inclusion of women as participants in biomedical research. In 1993, the US Food and Drug Administration reversed its 1977 policy and allowed women of childbearing age to participate in research studies, substantially increasing the pool of women eligible for inclusion in randomized clinical trials [6]. Likewise, the National Institute of Health (NIH) enacted a new policy requiring women and minorities to be included in research [7]. In 2000, the Canadian government established the Institute of Gender and Health as one of the 13 national research institutes that comprise the Canadian Institutes of Health Research. Similarly, The European Union instituted Sex and Gender Equity in Research (SAGER) guidelines advise including a discussion of whether or not sex and gender are relevant to a given line of investigation [8]. The SAGER guidelines, as well as questions included in the research application process, set a precedence to include similar guidelines in the NIH application review process. In fact, since 2015, investigators seeking NIH funding must provide justification for choosing to study only one sex. This is particularly critical if there are known sex differences in the area under investigation.

Despite these achievements, the number of studies designed and conducted in a manner to address sex differences remains in the minority. Given the FDA’s recommended change in zolpidem dosage for women occurred only 4 years ago, there are likely to be other medications presently marketed to both males and females for which there are relatively little data regarding efficacy and safety by sex. Moreover, many pharmacologic agents are first studied in cell culture lines where the sex of the cells are not known or reported in the literature. The 2015 NIH policy statement released by the NIH Director, Dr. Francis Collins, and NIH Office of Research on Women’s Health Director, Dr. Janine Clayton, emphasized the importance of knowing the sex of cell lines and tissues so that the very basis of our science is informed by sex as a biological variable (SABV) [9].

There are several stages in the conduct of research, from hypothesis generation to federal funding, where consideration of SABV can be assessed and further encouraged. One such point is protocol development and submission to the ethics committees or institutional research review boards (IRB) that oversee research. Given the requirement that investigators consider sex and/or gender differences in their research or adequately justify why they are not planning to do so, in order to receive federal funding as well as publish their results in certain high-impact journals, we sought to determine the proportion of investigators at the University of Pennsylvania who currently consider sex and/or gender at the stage of protocol development and submission to the University’s IRB. We hypothesized that a minority of investigators will demonstrate knowledge of the importance of sex or gender considerations at the time of their research protocol development as the NIH mandate is new and many investigators do not feel equipped financially or scientifically to address SABV, gender, or both in their research. We focus, in this first stage of our investigation, on studies that have or will collect data from human participants, as NIH policies regarding the inclusion of women in clinical research have been established for decades. For the purposes of our discussion, one’s sex is determined by the presence and dosage of X or Y chromosomes, which in most cases will be XX (female) and XY (male). When we refer to gender, we are referencing the individual’s sense of themselves as being a man or woman, which is typically based upon the social and cultural constructs of their community [10, 11]. While progress in the consideration of sex and gender differences in health among cis-gender/cis-sex individuals has been slow, a focus on sex and gender in regard to the health of the transgender/transex community is non-existent and an area of growing importance.

## Methods

Research staff in the Penn Center for Women’s Behavioral Wellness and Penn PROMOTES Research on Sex and Gender in Health reviewed 240 human subject-related research protocols approved by the University of Pennsylvania IRB between January 1, 2015, and July 1, 2015. Protocols were accessed through the Human Subjects’ Electronic Research Application (HSERA) website with expedited approval from the University of Pennsylvania IRB. Funding source, type of data collected, general area of research, and population being proposed for the investigation were documented using an electronic database system (REDCap) [12]. Protocols were electronically searched for the terms “sex” or “gender,” and the section of the protocol including the term was reviewed to document the rationale for having selected one or more of these terms. Four reviewers split the protocols for initial review. However, a second reviewer randomly selected 20 protocols to re-review and noted that searching for the terms “sex” and “gender” was not always informative regarding the rationale for choosing to study a given population. Hence, a second reviewer processed all 240 protocols again, this time adding the terms “male” and “female” to the search. Again, rationale for inclusion of “males” or “females” alone or both was documented.

After drawing information directly from the IRB protocol, the condition under investigation was searched using PubMed to determine the degree of knowledge regarding sex and/or gender differences with respect to aspects of the said condition. The topic under investigation was entered into a PubMed field and co-searched with the terms “gender” or “sex” separately. Abstracts, and if necessary manuscripts, from the articles identified in the search were reviewed to make the determination regarding degree of sex or gender differences documented for a given condition. All PubMed searches were saved in REDCap. The focus of research was then rated on a scale from “0” indicating the literature suggests that there are no sex or gender differences to a “4” indicating the literature suggests that there is extensive evidence of sex or gender differences in the topic/condition under investigation (Table 1).

**Table 1:** Rating scale for evidence of sex/gender differences

Rating	Definition
PubMed literature review of the topic under investigation revealed….	
0	No evidence of sex or gender differences
1	Evidence of sex or gender differences, but studies are small, from only one lab, or not well controlled
2	Evidence of sex or gender differences in at least one aspect of the topic under investigation. Studies may be small but come from multiple research groups or have been replicated.
3	Evidence of sex or gender differences in at least two aspects of the topic under investigation. Studies may be small but come from multiple research groups or have been replicated.
4	Evidence of sex or gender differences in at least two aspects of the topic under investigation. Studies are large and from multiple groups.
NA	No research has directly studied sex/gender differences for this disorder or condition.

*NA* not available

Multiple subcategories (Table 2) were also rated under sex and gender. Subcategories were chosen to reflect, in general, the biological nature of sex and the sociocultural nature of gender. As with the topics under investigation, subcategories were rated on a scale of 0 to 4, from no evidence supporting sex or gender differences to very strong evidence supporting sex or gender differences. For both sex and gender, there was the additional option of “no literature is present to draw a conclusion.”

**Table 2:** Subcategories for sex and gender differences

Subcategories	
Sex differences	
Pathophysiology	
Risk for the disorder in question (i.e., prevalence in one sex greater than the other)	
Age at onset	
Type and severity of symptoms	
Treatment (efficacy and/or risk for adverse events)	
Gender differences	
Risk for the disorder in question (i.e., prevalence in one sex greater than the other)	
Behavioral risk factors associated with the disorder/condition	
Treatment seeking and utilization of services/procedures for the condition	

“Risk for the disorder in question” was included as a subcategory under sex and gender differences as both sex and gender may contribute to the prevalence of disorder/condition

## Results

### Characteristics

Table 3 displays the general characteristics of the protocols reviewed. A total of 240 protocols were included. The majority of the protocols were applying for a full review (87.1%), with 10.8% applying for an expedited review and 2.1% applying for an exempt review. The majority of protocols indicated that both males and females were to be studied (85.4%), though use of one of the key search terms did not occur in all cases. While funding source varied, only 23.8% of the protocols had no funding source and the other sector accounting for the most funding was pharmaceutical (37.9%). The majority of protocols proposed research within the areas of oncology (30.4%), medicine (29.2%), or behavioral health (19.2%). Of the oncology-related protocols, 60.3% focus on topics that were found (according to PubMed search) to have at least a moderate sex difference, gender difference, or both. Of the 240 protocols, only 45 (18.8%) mentioned the keywords “sex” or “gender.” However, 165 protocols (68.8%) mentioned at least one of the following: “male,” “female,” “men,” “women,” “sex,” or “gender”. The most common (86.1%) location for mentioning one of these key terms was in the inclusion/exclusion section of the protocol. In contrast, only 7.3% of the protocols mentioned one or more of these key terms in the study design section.

**Table 3:** General characteristics of IRB protocols

	Number of protocols *N* (%)
	(*n* = 240)
Population proposed for investigation	
Males	8 (3.3)
Females	26 (10.8)
Both	205 (85.4)
Not applicable	1 (0.4)
Type of IRB review	
Full	209 (87.1)
Expedited	26 (10.8)
Exempt	5 (2.1)
Type of data to be studied	
New data	231 (96.3)
Existing population database	9 (3.8)
Funding source	
No funding	57 (23.8)
National Institutes of Health	30 (12.5)
Foundation	15 (6.3)
Penn Internal Grant or Funds	12 (5.0)
Pharmaceutical	91 (37.9)
Other funding	42 (17.5)
Mention of keywords	
Mention “sex” or “gender”	45 (18.8)
Mention “male,” “female,” “men,” or “women”	155 (64.6)
Mention at least one of the following: “sex,” “gender,” “male,” “female,” “men,” and “women”	165 (68.8)
Area of investigation	
Behavioral/neurological/psychological	46 (19.2)
Medicine	70 (29.2)
Surgery	35 (14.6)
Oncology	73 (30.4)
Obstetrics and gynecology	9 (3.8)
Genetics	4 (1.7)
Pediatrics	3 (1.3)
Location of keywords within protocol	
	(*n* = 165)
Objectives	17 (10.3)
Background	19 (11.5)
Study design	12 (7.3)
Populations	32 (19.4)
Inclusions/exclusions	142 (86.1)
Procedures	8 (4.8)
Other	6 (3.6)

### Evidence of sex and gender differences

Table 4 displays the number (proportion) of protocols ranked according to the level of known sex differences and gender differences as determined by the PubMed literature review. No relevant literature was available for 17.9% of the protocols, leaving these unranked with respect to evidence of sex or gender differences. For 19.2% of the protocols, the literature indicated no or minimal sex differences or the possibility of sex differences, but contradictory evidence. The majority of protocols, 62.9%, focused on topics for which the literature supported at least moderate (rated 2 or higher) sex differences in at least one aspect of the disease/condition being investigated.

**Table 4:** Ratings of protocols for sex and gender differences in area of investigation according to results of PubMed searches

	Number of protocols (*n* = 240)
	*n* (%)
Evidence of sex differences	
0, no sex differences	15 (6.3)
1, there may be sex differences	31 (12.9)
2, sex differences in at least 1 aspect	61 (25.4)
3, sex differences in at least 2 aspects	56 (23.3)
4, sex differences in at least 2 aspects, studies are large, from multiple groups	34 (14.2)
NA, no literature available	43 (17.9)
Evidence of gender differences	
0, no gender differences	43 (17.9)
1, there may be gender differences	51 (21.3)
2, gender differences in at least 1 aspect	52 (21.7)
3, gender differences in at least 2 aspects	30 (12.5)
4, gender differences in at least 2 aspects, studies are large, from multiple groups	13 (5.4)
NA, no literature available	51 (21.3)

Scientific focus of each protocol was reviewed in PubMed for evidence of research indicating sex or gender differences exist in pathophysiology, phenomenology, clinical course, or treatment

For 21.3 and 39.2% of the protocols, there was no or minimal respectively relevant literature available to judge the presence of gender differences in the topic under investigation. There was evidence of at least moderate gender differences in the topics of investigation under study in 39.6% of the protocols.

### Rationale for mentioning keywords

Table 5 displays the protocols separated by their ratings for sex and gender differences to show the manner in which the keywords were used within the protocols. Sixty-five percent (*n* = 156) of the protocols were studying topics with significant sex differences, significant gender differences, or significant differences in both (scored 2, 3 or 4 per Table 1). Of these protocols, 46.2% mentioned the keywords (“sex,” “gender,” “male,” “female,” “men,” or “women”) in the inclusion/exclusion criteria regarding pregnancy or the potential to become pregnant. Additionally, 29.5% of these protocols mentioned the keywords in reference to the population that would be studied (e.g., “This study will recruit males and females”). Only 4.5% of these protocols mentioned these words as a part of the rationale for choosing their particular study population, and 1.9% of these protocols mentioned these keywords in reference to possible impact on primary outcomes.

**Table 5:** Rationale for mentioning keywords

	Protocols studying topic with significant sex AND/OR gender differences *n* = 156 (%)	Protocols studying topic with no or little evidence of sex or gender differences or have not been studied *n* = 84 (%)
Possible impact on primary outcomes	3 (1.9)	2 (2.4)
Rationale for choosing particular study population	7 (4.5)	17 (20.2)
Inclusion/exclusion criteria, reason not specified	5 (3.2)	4 (4.8)
Inclusion/exclusion criteria, regarding pregnancy	72 (46.2)	29 (34.5)
Stating the population that will be studied	46 (29.5)	16 (19.0)
Data that will be collected or recorded, reason not specified	5 (3.2)	3 (3.6)
Provides background	10 (6.4)	5 (6.0)
Other	7 (4.5)	5 (6.0)

The 156 protocols represent those that focus on topics for which PubMed search indicates at least moderate sex difference, moderate gender difference, or both. Protocols rated “2” or higher (as described in Table 1) were considered to have moderate evidence

Thirty-five percent of the protocols were studying topics for which the literature review found evidence of no sex or gender differences, found little evidence of sex or gender differences, or found that there was no literature available to rate sex or gender differences (scored 0, 1 or NA per Table 1). Of these protocols, 34.5% mentioned the keywords in the inclusion/exclusion criteria, regarding pregnancy. Either pregnant women were being studied or pregnant women were excluded. Additionally, 20.2% used these keywords when describing the rationale for choosing the particular study population. However, only 2.4% of these protocols mentioned these keywords in order to find the possible impact of sex or gender on primary outcomes.

## Discussion

Historically, sex and gender are factors that have been overlooked in clinical and preclinical research. Currently, policies exist to enhance participation of women in clinical research, and while the female/male participant ratio has increased over the past three decades, few studies report outcomes by sex. This practice is particularly disconcerting with respect to randomized clinical trials given the evidence that female sex is a risk factor for side effects and adverse events related to medication treatment [13–15]. For many chronic medical conditions, such as migraines [16, 17], autoimmune disease [18, 19], cardiovascular disease [20, 21], and dementia [22–24], sex contributes to the prevalence, symptom presentation, and clinical course. The contribution of gender to health and healthcare is evident in the behavioral factors that contribute to risks for a disorder and the propensity to seek evaluation and intervention. Despite the evidence for at least moderate sex and/or gender differences in 65% of the topics proposed to the University of Pennsylvania IRB for investigation, the vast majority of these protocols (98%) did not mention consideration of sex or gender as potential outcome modifiers. The sex of the individuals under investigation was most frequently mentioned in the context of either including or excluding pregnant women. While exclusion of pregnant women may be critical for participant safety, it does not indicate investigator awareness of other important sex/gender factors in their research.

Forty-six percent of the protocols indicated that the study had no funding and was supported by an internal pilot funding mechanism or “other funding.” Typically, the goal of these early stage investigations is to obtain pilot data to support federal funding applications in the years to come. As these are all protocols for studies, which are currently underway, there is concern that the investigations will not yield preliminary findings to address the new NIH mandate at the time of application for federal funding. In some cases, such as the 38% of studies reported to be associated with pharmaceutical funding, the Penn investigator may be bound to an IRB protocol that is standardized across research sites. Future research should examine the nature of pharmaceutical funded studies (i.e., investigator initiated or multi-site clinical trials) as the capacity for a given investigator to address SABV varies.

These data indicate that the current system, which is likely not specific to the University of Pennsylvania, lacks a consistent systematic approach for studying and reporting on sex and gender within research. It is not necessarily the case that all studies need to include sex or gender the statistical model, but the NIH mandate to justify the use of one sex requires a thoughtful response on the part of the investigator. One possible stage in the process of clinical research at which investigators could be encouraged to learn more about the role of sex and gender in their areas of research would be at the IRB protocol submission stage. We propose that requiring investigators to address SABV in their research at the time of IRB protocol submission would not only increase awareness of these factors at the time of study design and development of data analytic plan but would also be consonant with the role of the IRB in ensuring research quality and safety.

In addition, there has been some effort on the part of editorial boards for high-impact scientific journals to require that authors are clear about the sex of the organism under investigation. The effectiveness of journal policies was demonstrated in 2005, when the International Committee of Medical Journal Editors (ICMJE) implemented the policy that all medical journal editors require manuscripts describing randomized clinical trials provide evidence of having registered the trial with a public trials registry, such as ClinicalTrials.gov. This change in policy leads to a 73% increase in registered trials in the 4 months surrounding the implementation [25]. However, many journals remain unsure or unwilling to incorporate sex and gender guidelines into their recommendations for authors. According to a survey conducted by the European Association of Science Editors’ Gender Policy Committee, only 7% of respondents reported that their journals had existing policies with respect to sex and/or gender for their publications [26].

The journals that have embraced the importance of reporting sex, gender, or both with respect to participants, animals, and tissue/cells emphasize consideration in the data analytic plan and results [27, 28]. Then, there are journals such as *Biology of Sex Differences* that focus specifically on sex and gender as critical biological or sociocultural factors, respectively, that must be considered in order to be accepted for review. The journal *Endocrinology* (Endocrine Society) includes the following in their instructions to the authors regarding the reporting of sex of research subjects, “The strain (when appropriate) and sex of animals must be indicated. If both males and females were used, the numbers of animals from each sex must me indicated, and it must be indicated whether the sex of the animal was considered a factor in the statistical analysis of the data”. (https://academic.oup.com/endo/pages/Author_Guidelines),

A recent editorial in *The Lancet* proposed guidelines on reporting sex and gender in medical journals. Briefly, guidelines for journal editorial boards included (1) requiring the correct use of the terms sex and gender, (2) requiring analyzing the data by sex, gender, or both of study participants, animals, and tissues/cells (when appropriate), (3) analyzing the influence of sex, gender, or both on results where appropriate, and (4) requiring that post hoc consideration of sex or gender be interpreted cautiously [29]. The instructions to authors wishing to publish in the *The Lancet* are as follows: “We encourage researchers to enroll women and ethnic groups into clinical trials of all phases, and to plan to analyze data by sex and by race.” While we welcome additional journal editorial boards to create policies related to sex and gender for the articles they publish, it is rather late in the process of research for these policies to be fully impactful. Again, we argue that the point of greatest potential impact on future research would be to require the consideration of sex and gender at the IRB submission stage when choice of study population and recruitment and data analytic plans are still relatively flexible.

However, adding a set of questions to the IRB approval process has some obvious disadvantages. At most institutions, service on the IRB is voluntary and time-consuming for faculty who must review multiple protocols each week. Protocol topics may be outside the reviewer’s immediate area of expertise making it difficult to determine whether the investigator has adequately addressed sex, gender, or both in the proposed project. Instituting such a policy would require that the IRB staff and faculty are educated regarding the NIH policies as well as what is a sufficient justification for selecting to study one sex or the other. Reviewers would need to be savvy in their critique of the subject recruitment and data analytic plans for those projects, which propose to study both sexes. Finally, the investigators themselves may view this new requirement as just “one more hurdle” in the progress of research. Many of these concerns could be addressed with thoughtful, brief, education, and training sessions for IRB staff and faculty. Faculty investigators from research areas for which sex or gender issues are prominent could be provided “in laboratory” workshops focusing on the new policy in relationship to their specific areas of research. At the University of Pennsylvania, this process is the responsibility of a new school-wide initiative, Penn PROMOTES Research on Sex and Gender in Health, which is supported by the Office of the Vice Dean for Inclusion and Diversity at the Perelman School of Medicine and the Office of the Vice Provost for Research. In order to insure consistency in considering sex and gender during the review process conducted by IRB review boards at academic institutions across the nation, guidance from the Office for Human Research Protections and the FDA may be required.

In summary, this survey was limited to a subset of the clinical research being conducted a one major research institution. While the Penn IRB protocol template requires investigators to provide a detailed statistical approach for primary and secondary analyses, we cannot rule out that investigators have intentions to consider SABV or gender impact on outcomes that were not included in the IRB protocol. The protocols reviewed may not be representative of the entire year of protocol submissions, though we did not observe a shift in consideration of sex, gender, or both over the block of time represented by the protocols reviewed. We propose that findings are likely to be similar at other institutions and challenge them to consider policies to enhance the consideration of sex and gender in their biomedical, behavioral research endeavors.

## Conclusions

Consideration of SABV and gender impact in biomedical research is critical to the conduct of the most rigorous science and is no longer an option for investigators who seek federal funding. We propose that requiring investigators to report and justify their plans with respect to subject recruitment, use of human products and materials, and secondary analysis of previously collected data at the time of IRB protocol submission will enhance research quality and health for both men and women.

## Abbreviations

FDA: Food and Drug Administration
HSERA: Human Subjects’ Electronic Research Application
ICMJE: International Committee of Medical Journal Editors
IRB: Investigational review board
NIH: National Institutes of Health
SABV: Sex as a biological variable
SAGER: European Union instituted Sex and Gender Equity in Research
